# Secreted Osteoclastogenic Factor of Activated T Cells (SOFAT) Is Associated With Rheumatoid Arthritis and Joint Pain: Initial Evidences of a New Pathway

**DOI:** 10.3389/fimmu.2020.01442

**Published:** 2020-07-28

**Authors:** Marcelo Henrique Napimoga, Weslley Danny Dantas Formiga, Henrique Ballassini Abdalla, Carlos Antônio Trindade-da-Silva, Camila Motta Venturin, Elizabeth Ferreira Martinez, Ana Carolina Rossaneis, Waldiceu A. Verri, Juliana Trindade Clemente-Napimoga

**Affiliations:** ^1^Laboratoy of Neuroimmune Interface of Pain Research, Faculdade São Leopoldo Mandic, Instituto de Pesquisas São Leopoldo Mandic, Campinas, Brazil; ^2^Faculdade São Leopoldo Mandic, Instituto São Leopoldo Mandic, Campinas, Brazil; ^3^Departamento de Ciências Patológicas, Universidade Estadual de Londrina, Londrina, Brazil

**Keywords:** inflammation, SOFAT, rheumatoid arthritis, pain, osteoclast

## Abstract

Rheumatoid arthritis (RA) has an inflammatory milieu in the synovial compartment, which is regulated by a complex cytokine and chemokine network that induces continuously degenerative and inflammatory reactions. The secreted osteoclastogenic factor of activated T cells (SOFAT) is a unique cytokine and represents an alternative pathway for osteoclast activation. In this study, we examined whether SOFAT is able to induce joint pain and investigated the presence of SOFAT in a Collagen-induced Arthritis (CIA) model and in human subjects. Here, we found that an intra-articular stimulation with SOFAT (1, 10, 100, or 1,000 ng/10 μl) in the knee joint significantly decreases the mechanical threshold in the hind paw of mice (*p* < 0.05). Moreover, after a second injection of SOFAT, the mechanical threshold decrease was sustained for up to 8 days (*p* < 0.05). In the CIA model, the immunohistochemical assay of knee joint showed positivity stained for SOFAT, and the mRNA and protein expression of SOFAT were significantly higher in the affected-group (*p* < 0.05). Besides, the mRNA of RANKL, IL-1β, IL-6, and IL-15 were significantly higher in the affected-group (*p* < 0.05). Finally, SOFAT was detected in the synovial fluid of RA patients, but not in OA patients (*p* < 0.05). In conclusion, SOFAT is up regulated in inflammatory milieu such as RA but not in non-inflammatory OA. SOFAT may be a novel molecule in the complex inflammatory phenotype of RA.

## Introduction

The term arthritis is used to refer to a group of diseases that affect the joints with varied degrees of inflammation in sterile and septic forms (1, 2). According to the Centers for Disease Control and Prevention (CDC) and Arthritis Foundation, around 54 million people have any kind of arthritis in the USA. Despite the well-recognized burdens of arthritis, such as pain, swelling, and stiffness, 60% of arthritis-affected report limitations in their daily activities (3). The decline in quality of life as well as the risk of comorbid illness (obesity, diabetes, and heart disease) represent an individual and society public cost since a high percentage of occurrence is in the working-age population (18–64 years old) (4, 5). Within this set of diseases commonly called arthritis, osteoarthritis (OA) represents the highest incidence; however, rheumatoid arthritis (RA), gout, and fibromyalgia are equally relevant (6).

The immune system fails to recognize the self in RA. The continuously degenerative and inflammatory reactions result from an abnormal innate and adaptive immune responses primarily affecting the joints but also extra-articular tissues (7). Failure of therapy treatment could lead to an irreversible stage of joint destruction and consequently disability of daily activities (8). Intense joint pain is a clinical symptom that profoundly decreases the quality of life of RA patients (9). OA is a degenerative disease (10) primarily characterized by progressive cartilage degeneration and secondarily by synovial inflammation. Although OA was initially considered as a non-inflammatory disease, associated especially with obesity, evidence demonstrates an important role of the chondrocytes phenotype in cartilage breakdown (10). In summary, RA or OA present destruction of cartilage and underlying bone despite their different etiologies and biological mechanism.

In this scenario, activated T cells stood out for their modulatory activity of bone turnover and by representing an important source of osteoclastogenic cytokines (11), including in RA (12, 13). Moreover, T cells induce osteoclast formation through RANKL (receptor activator of nuclear factor kappa-B ligand) and TNF-α (tumor necrosis factor-α) (14). Also, IL-1 and IL-6 (interleukin) are released by T cells, leading to RANKL expression by osteoblast (15). IL-15 recruits and activates T cells, which will produce TNF-α in RA. TNF-α, IL-1β, IL-6, and IL-15 induce pain in RA (16, 17).

A novel activated human T-cell-secreted cytokine, named as the secreted osteoclastogenic factor of activated T cells (SOFAT), has been characterized (11). Interestingly, SOFAT can induce bone loss in a RANKL-independent manner in periodontal disease (18, 19) as well as directly stimulate IL-6 by human osteoblasts (11), modulate osteoblast activities sustaining the inflammatory environment (20), and, finally, represent a putative marker for osteoclast in bone lesions (21).

In the present study, we examined whether SOFAT could induce joint pain. Other than this, we investigated the presence of SOFAT in collagen-induced arthritis (CIA) model. We therefore investigated whether the increase of SOFAT is an observed factor in patients previously diagnosed with RA or OA.

## Methods

### Animals Approved and Care

Male Swiss mice (25–30 grams) and male DBA/1J mice (6–9 weeks) were used in this study. Animals were randomly assigned and housed in plastic cages (*n* = 5/per cage) in a temperature-controlled room (23 ± 1°C) at a 12:12 light cycle with access to water and food *ad libitum*. All animal experiment procedures and protocols were approved by the Committee on Animal Research of Faculdade São Leopoldo Mandic, Brazil (No. 2019/019) or Committee on Animal Research of Universidade Estadual de Londrina (approval under the number CEUA-UEL 28016.2014.91) and are in accordance with the guidelines by the National Council for Control of Animal Experimentation (CONCEA), ARRIVE (22), and the International Association for the Study of Pain (IASP) for the study of pain in conscious animals (23). All efforts were made to minimize animal suffering and to reduce the number of animals used. Each experimental group had a number of six animals in each experiment, and all experimental procedures were repeated twice.

### Mechanical Hyperalgesia

A mechanical hyperalgesia test was performed by an electronic version of von Frey's filaments, as previously described (24). During the light phase (between 9:00 a.m. and 5:00 p.m.), the test was carried out in a quiet, temperature-controlled room. Mice were placed in acrylic cages with wire grid floors 30 min before the experiment for acclimatization with the new environment. The assessment of knee joint hyperalgesia in the femur–tibial joint consist of an electronic pressure meter with a force transducer fitted with polypropylene tip (Insight instruments, Ribeirão Preto, SP, Brazil). The articular pain was evaluated with a large tip (4.15 mm^2^) due to the exclusion factor of cutaneous nociception. A progressive pressure was applied to a central area of plantar surface of hind paw to induce flexion of the femur–tibial joint and subsequent hind paw withdrawal. The pressure intensity of the withdrawal threshold (in grams) was automatically recorded. Investigators were blinded to the groups.

For this study, mice were treated with an intra-articular injection on knee joint, in different concentrations of SOFAT (1, 10, 100, or 1,000 ng/10 μl) as a noxious challenge or vehicle (saline 10 μl). The intensity of mechanical hyperalgesia at the intervals of 1, 3, 5, 7, 24, 48, 72, 96, and 120 h after the stimulus was evaluated. A two-injection assay was carried out. The first injection was performed in the baseline period (0 days), and the second injection on the fifth day. The mechanical hyperalgesia was then evaluated daily for 10 days.

### Collagen-Induced Arthritis (CIA) in DBA/1J Mice

CIA protocol was induced in mice as previously described by Su and collaborators (25). Concisely, male DBA/1J mice (6–9 weeks) were immunized intradermally at 1.5 cm from the tail base with 100 μg of chicken sternal hyaline Collagen type 2 (CII) (Sigma, #C9301), dissolved in 100 μl acetic acid (0.05 mol/l), and mixed with an equal volume of Freund's Complete Adjuvant (CFA) (Difco Laboratories, Detroit, MI, USA). Twenty-one days later, animals were boosted with 100 μg of CII and emulsified in incomplete Freund's adjuvant (IFA). Mice were examined daily for signs of arthritis as described (25). By the end of 15 days, all groups were anesthetized with xylalizine and ketamine intraperitoneal and sacrificed by cervical dislocation. Knee and paw joints were collected for histological analyses, western blot, and RT-qPCR.

### Subjects

Seven individuals diagnosed with rheumatoid arthritis (RA) (*n* = 4) or with osteoarthritis (OA) (*n* = 3), were selected from the General Hospital of the Medical School of Ribeirão Preto of the University of São Paulo (HCFMRP-USP). The subjects with an indication for articular puncture were invited to participate of this study. All admitted subjects signed their informed consent. The Ethics Committee in Human Research at Ribeirão Preto General Hospital and Ribeirão Preto Medical School (protocol 38156414.4.0000.5374) previously approved this study protocol.

### Synovial Liquid Collection

Patients previously diagnosed with AR or OA who also displayed indications of an articular puncture in the knee joint were selected. The procedure was performed under aseptic and antiseptic patterns (Povidone-iodine, PVP-I). The intra-articular puncture was performed with a 10 ml syringe coupled with a 25 × 7 needle. The synovial liquid was immediately stored in ice until the sample processing could occur.

### Sample Preparation and Protein Extraction

Knee and paw samples for Western Blotting analysis (CIA protocol) were homogenized in 500 μl of the appropriate buffer containing protease inhibitors (Ripa Lysis Buffer, Santa Cruz, Biotechnology, Dallas, Texas, USA) using a specific sample homogenizer (BeadBlaster™ 24, Benchmark, Triple-Pure High Impact Zirconium Beads, Beads, Ø: 1.0 mm). After four cycles of 30 s with resting period of 40 s, the samples were centrifuged for 10 min/10,000 rpm/4°C. The total amount of extracted proteins was colorimetrically measured using the micro bicinchoninic acid (BCA) protein assay kit (Thermo Scientific, Rockford, IL, USA). The supernatants were stored at −20°C until further analysis.

Samples used for quantitative PCR (knee and paw joint mice; CIA protocol) were homogenized in TRIzol® reagent (ThermoFisher, MA, USA) with the homogenizer as previously detailed. Total RNA was extracted according to manufacturer's directions and stored at −70°C. The RNA concentration for each sample was determined by optical density using a micro-volume spectrophotometer (Nanodrop 1000, Nanodrop Technologies LLC, Wilmington, NC, USA).

Synovial liquid samples were immediately stored in ice after the intra-articular puncture. At the end of the surgical procedure, samples were centrifuged for 5 min/10,000 rpm/4°C, and the supernatant was collected and stored. The protein concentrations were determined using the bicinchoninic acid protein assay kit (Thermo Scientific, Rockford, IL, USA).

### Reverse Transcription and Quantitative Polymerase Chain Reaction (RT-qPCR)

Reverse transcription of total RNA to cDNA was performed using DNase (Turbo DNA-frees, Ambion Inc., Austin, TX, USA). The reaction was carried out using the First-Strand cDNA synthesis kit (Roche Diagnostic Co., Indianapolis, IN, USA) following the manufacturer's recommendations. The qPCR was performed using GoTaq® 2-Step RT-qPCR System (Promega Corporation, WI, USA) and specific primers (Applied Biosystems®, ThermoFisher, MA, USA). The mRNA level of glyceraldehyde 3-phosphate dehydrogenase (GAPDH) was used as a reference gene.

### Western Blotting

Equal amounts of protein (30 μg) from human synovial liquid or mice samples were loaded onto 10% SDS/PAGE gel and transferred to a nitrocellulose membrane. The membranes were blocked in TBS (20 mM Tris-HCl, 150 mM NaCl, and 1% Tween 20, pH 7.53) containing 5% non-fat dry milk for at least 2 h. Membranes were incubated with specific primary antibodies—anti-SOFAT (Rheabiotech), 1:500, overnight and GAPDH (Cell Signaling), 1:1,000, for 2 h. After incubation period, the membranes were rinsed with TBS, and subsequently incubated for 1 h with the secondary antibody, peroxidase-conjugated anti-rabbit IgG (Vector Laboratories, Burlingame, CA, USA). The bands of the membranes were visualized using the enhanced chemiluminescence (ECL) solution for 3 min (Amersham Biosciences, Piscataway, NJ, USA), and the digital image was obtained by CCD camera imaging for chemiluminescence (ImageQuant LAS 4000 mini, GE Healthcare Life Sciences, Pittsburgh, PA, USA). The program Image J (National Institutes of Health, Bethesda, USA) was applied to measure the optical density of the bands.

### Enzyme-Linked Immunosorbent Assay (ELISA)

Levels of SOFAT were quantified in capture enzyme-linked immunosorbent assays (ELISA) as previously described (19). Briefly, microtiter plates (Costar 3590, Corning, NY) were incubated for 24 h at 4°C with 5 μg/ml of rabbit IgG anti-human SOFAT in carbonate–bicarbonate buffer, pH 9.6, following the manufacturer instructions. All antibody reagents were affinity purified and obtained from (Rheabiotech Laboratory, Campinas, SP, Brazil). After incubation period, plates were washed and blocked for 1 h at room temperature with 0.1% of BSA (bovine serum albumin). The washed process was repeated, and synovial liquid samples were pipetted. The plate was incubated for 2 h at room temperature. The assay included serial dilutions (500, 250, 125, 62.5, 31.25, 15.62, 7.81, and 3.90 lg/ml) of a standard sample of human SOFAT antibody (Rheabiotech). The secondary antibody was biotin-conjugated rabbit IgG anti-human SOFAT (Rheabiotech) at a dilution of 1:1,000. After incubation with a solution of avidin–peroxidase (30 min) at room temperature, a new series of washes was performed and substrate solution (TMB) was added and incubated for 15 min. The reaction was stopped with Stop Solution and read immediately in a spectrophotometer (Epoch, Biotek, Winooski, VT, USA) at 450 nm. Negative controls included an uncoated, no synovial liquid sample and no primary antibody wells. The total amounts of SOFAT were determined in nanograms per ml of synovial liquid sample. The assay was carried out in a blind fashion.

### Histological Sections

For histological analysis, the knee joints of mice (CIA protocol) were used. The samples were fixed in 10% buffered neutral formalin (48 h) and decalcified in a solution of 10% EDTA. Knee samples were washed in running water, dehydrated, and embedded in paraffin wax. For hematoxylin-eosin staining, sections of 3 μm were sliced and dyed. For immunohistochemical staining, a previously standardized protocol was used as reference (21). Briefly, samples sections (3 μm) were incubated with primary antibody of anti-SOFAT (1:300; Rheabiotech), followed by EnVision polymer HRP and Envision+ (Dako, SA, Denmark) for 1 h at 37°C. Ten minutes were used for developing at 37 with 3.3′-diaminobenzidine tetrahydrochloride and counterstained with Mayer hematoxylin (3 min; room temperature). Digital photomicrographs were taking from representative areas (Zeiss Axioskop 2 plus microscope, Carl Zeiss, Gottingen, Germany).

### Statistical Analysis

The statistical analyses were performed using a software program (GraphPad Prism 6.0, La Jolla, CA, USA). To determine if there were significant differences (*P* < 0.05) among groups, the data were analyzed using the Student *t*-test. Data are presented in figures as mean ± standard deviation (SD).

## Results

### SOFAT Induce Mechanical Hyperalgesia in the Knee Joint

**Figure 1:**
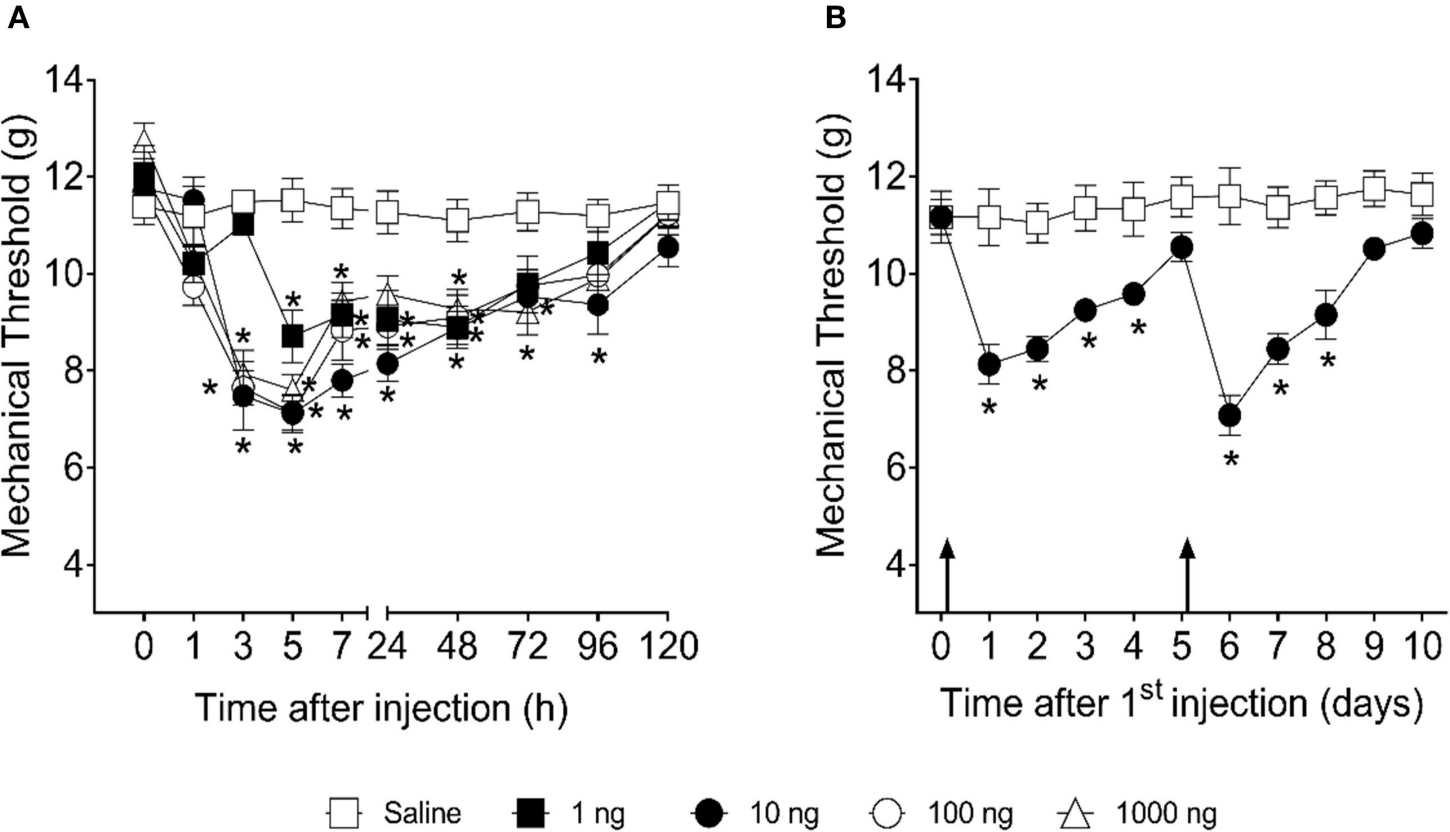
SOFAT inhibits mechanical hyperalgesia in mice. Mice received intra-articular stimulation with SOFAT (1, 10, 100, or 1,000 ng/10 μl) and mechanical hyperalgesia was evaluated at the intervals of 1, 3, 5, 7, 24, 48, 72, 96, and 120 h after the stimulus **(A)**. The effect of two intra-articular injections of SOFAT (10 ng/10 μl) on mechanical hyperalgesia was evaluated daily for 10 days. Mice received the first injection immediately after baseline measurement (Time 0) and the second injection immediately after the fifth day measurement, as indicated by the arrows **(B)**. Results are expressed as mean ± SEM (*n* = 6 per group per experiment, representative of two experiments). The symbol (*) means difference compared to saline group (*P* < 0.05: Two-way ANOVA, Tukey's test).

First, we investigated whether intra-articular injection of SOFAT in the knee joint could induce mechanical hyperalgesia in mouse (Figure 1). For that, titrated doses of SOFAT (1, 10, 100, or 1,000 ng/10 μl) were injected in the knee joint. Mechanical hyperalgesia was evaluated at the intervals of 1, 3, 5, 7, 24, 48, 72, 96, and 120 h after the stimulus (Figure 1A). We demonstrated here that intra-articular injection 1 and 10, 100, and 1,000 nanograms of SOFAT induce mechanical hyperalgesia 5 and 3 h later, respectively (Figure 1A). The hyperalgesia lasted until 96 h when the mechanical threshold returned to normal values. In a repetitive injection protocol, SOFAT induced hyperalgesia over 4 days upon the first administration, and, on the fifth day in which the hyperalgesia vanished, an additional administration of SOFAT induced enhanced hyperalgesia, which lasted up to the eighth day (*p* < 0.05; Figure 1B).

### Collagen-Induced Arthritis Augments SOFAT Levels

**Figure 2:**
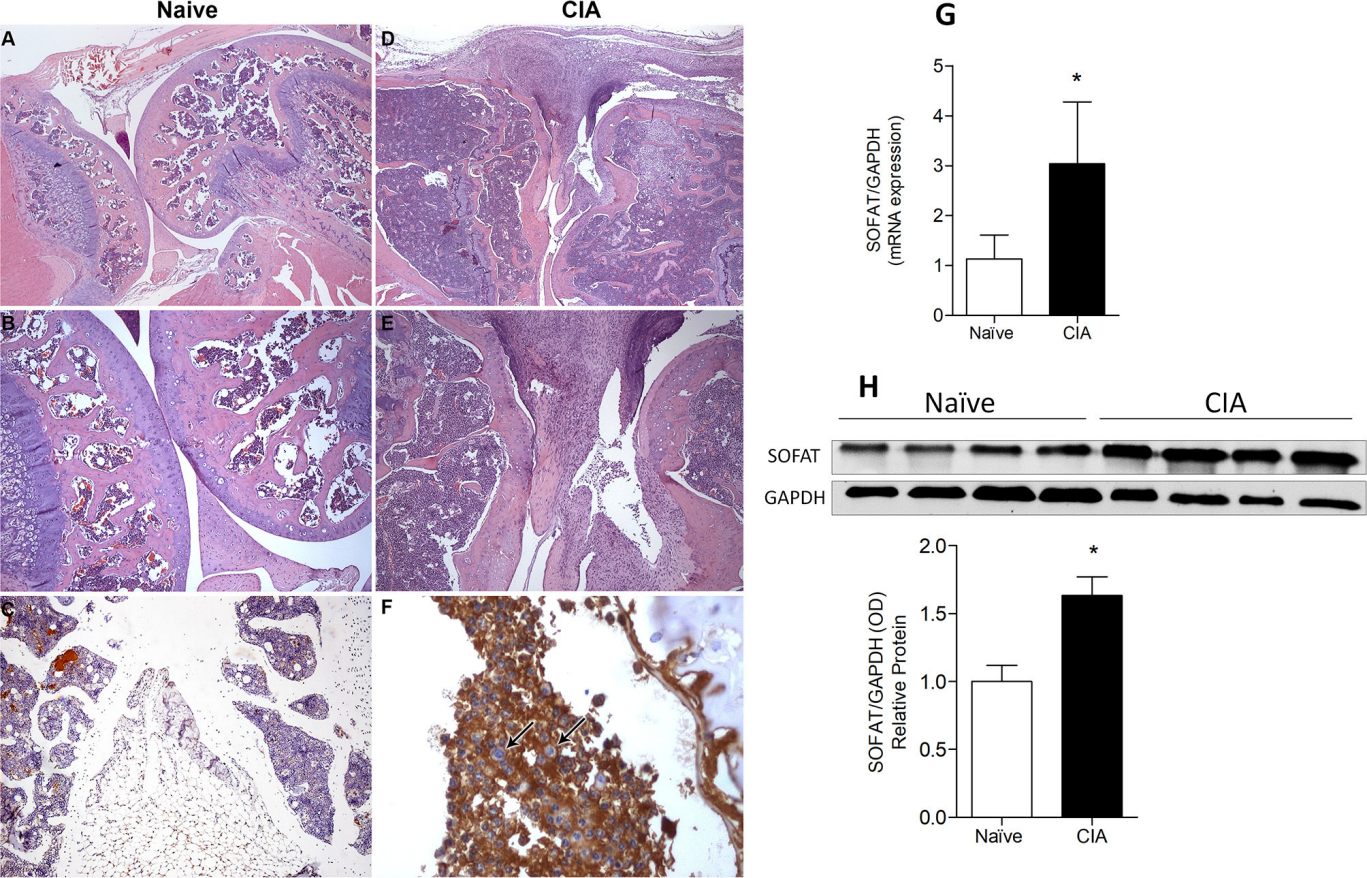
Collagen-induced arthritis showed a positive stain for SOFAT in the knee joint of mice. Histological sections of the knee joint were staining with hematoxylin-eosin (HE). Magnification of 20x **(A,D)** and 40x **(B,F)** were used. Images were taken from the naïve group, demonstrating no leukocyte infiltrate and bone or cartilage breakdown **(A,B)**. Images were taken from the CIA group showing vast leukocyte infiltrate and bone or cartilage disrupted **(D,E)**. Immunohistochemical staining of SOFAT in the knee joint. Magnification of 40x **(C)** and 40X **(F)** were used. The arrows demonstrate leukocytes in the inflammatory infiltrate into the synovium. Naïve group does not show positive stained for SOFAT **(C)** and the CIA group shown positive stained for SOFAT **(F)**. SOFAT mRNA expression in joints of mice induced or not by CIA protocol **(G)**. Representative bands of Western Blotting and protein fold changed **(H)** of SOFAT. The data are expressed as mean ± *SD* (*n* = 6 per group per experiment, representative of two experiments). The symbol (*) means mRNA or protein expression significantly higher than the naive group (*P* < 0.05: *T*-test, Tukey's test).

Given the capacity of SOFAT to induce joint pain, we next assessed the presence of SOFAT in a CIA model of arthritis. Histological sections with HE-staining were made in the knee joint of affected- or naive-group (Figure 2). Intense leukocyte infiltrates, pannus formation, cartilage, and bone breakdown were observed in the CIA model (Figures 2D,E) but not in the naive group (Figures 2A,B). Subsequently, immunohistochemistry for SOFAT exhibits high positive stain in the leukocyte infiltrate (Figure 2F). On the other hand, a weak or no stain was observed in the naive group (Figure 2C). In agreement with this, mRNA (Figure 2G) and protein expression of SOFAT (Figure 2H) showed statistical higher values (*p* < 0.05) in the CIA model when compared to the naive group.

### Collagen-Induced Arthritis Enhances Inflammatory and RANK/RANKL/OPG Axis

**Figure 3:**
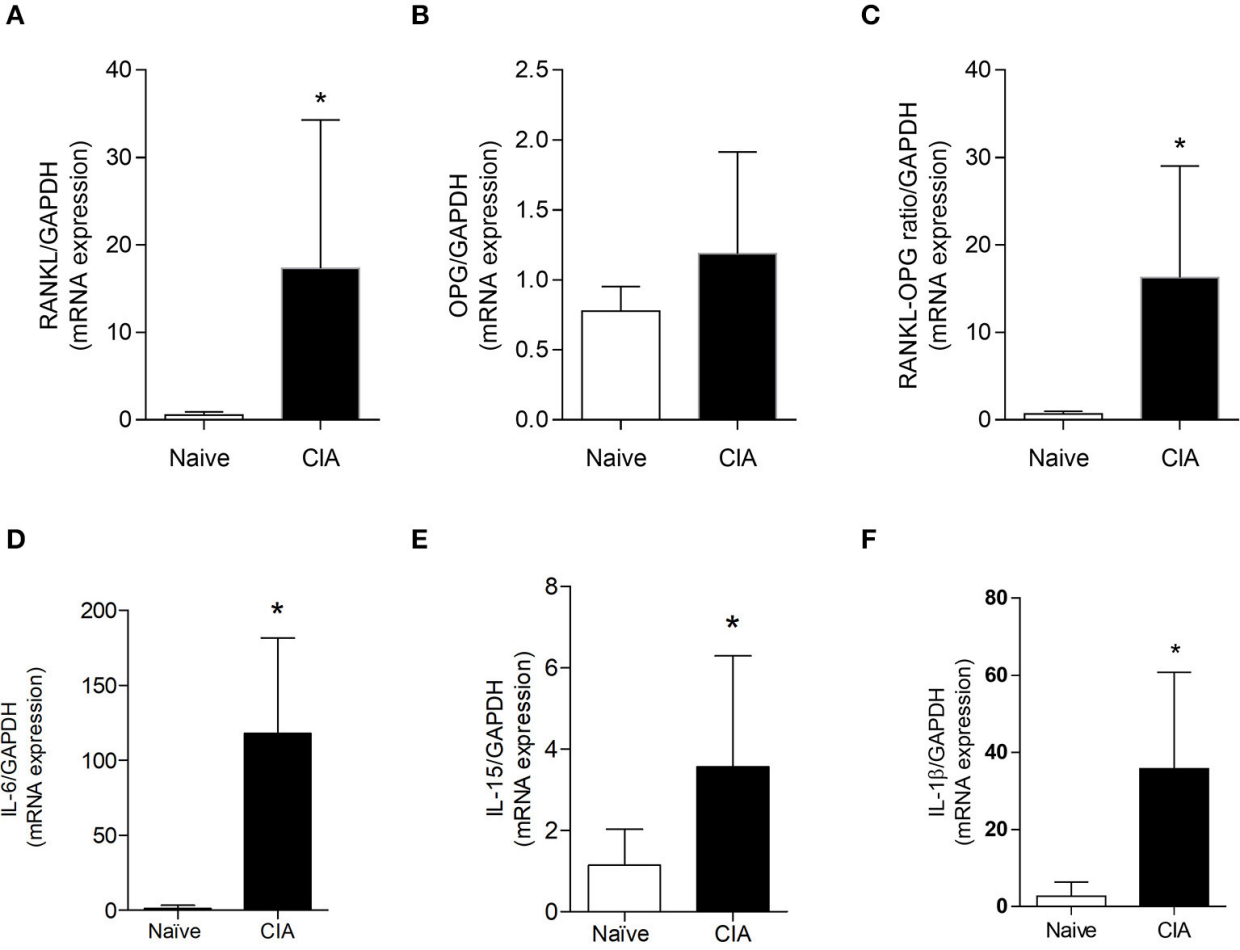
Collagen-induced arthritis enhances inflammatories and osteolytic markers. mRNA expression of RANKL **(A)**, OPG **(B)**, RANKL/OPG ratio **(C)**, IL-6 **(D)**, IL-15 **(E)**, and IL-1β **(F)** were performed in joints mice. The data are expressed as mean ± SD (*n* = 6 per group per experiment, representative of two experiments). The symbol (*) means mRNA significantly higher than the naive group (*P* < 0.05: *T*-test, Tukey's test).

Once it was demonstrated that CIA model augments the expression of SOFAT and leads to cartilage and bone breakdown, we investigated the presence of pro inflammatory mediators and RANK/RANKL/OPG axis markers (Figure 3). Our results showed a statistical increase (*p* < 0.05) in mRNA expression for RANKL (Figure 3A), RANKL/OPG ratio (Figure 3C), IL-6 (Figure 3D), IL-15 (Figure 3E), and IL-1β (Figure 3F) in affected groups when compared to the naive group. Only mRNA expression of OPG (Figure 3B) did not show a statistical difference between naïve and CIA groups.

### SOFAT Is Highly Expressed in Patients With Rheumatoid Arthritis (RA) but Not Osteoarthritis (OA)

**Figure 4:**
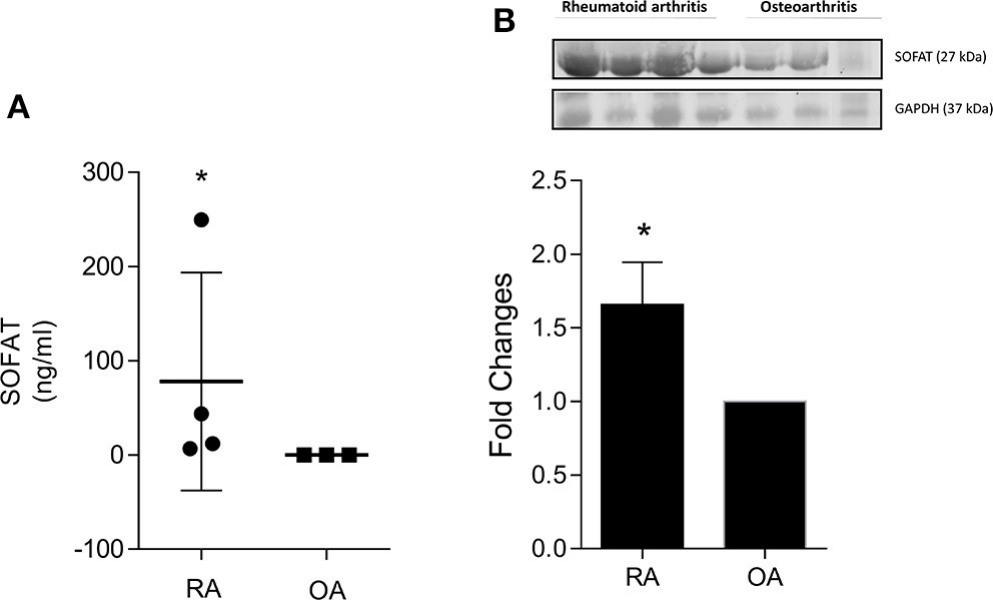
SOFAT is highly expressed in patients with Rheumatoid Arthritis (RA) but not Osteoarthritis (OA). The protein level of SOFAT **(A)** was analyzed in the synovial liquid of knee patients using ELISA. Representative bands and protein expression **(B)** of SOFAT were analyzed in the synovial liquid of knee patients using Western Blotting. The data are expressed as mean ± SD. The symbol (*) means protein level or expression significantly higher than the naive group (*P* < 0.05: *T*-test, Tukey's test).

Given the present animal model mimics RA, we investigated the presence of SOFAT in the synovial liquid of the knee joint of patients previously diagnosed with RA and OA (Figure 4). Our results demonstrated that the protein levels of SOFAT were significantly higher (*p* < 0.05) in patients diagnosed with RA than OA (Figures 4A,B).

## Discussion

Rheumatoid arthritis is a chronic inflammatory disease characterized by a vast lymphocyte infiltrate in the synovial membrane over the joints (8). RA probably arises from multiple hits, whereby an initial combination of lifestyle, environmental, and stochastic insults occurring in a genetically predisposed individual leads to breach of immunological tolerance (3). In the past decade, a new cytokine was described, namely, Secreted Osteoclastogenic Factor of Activated T cells (SOFAT), which is capable of inducing osteoclast formation in a RANKL-independent manner (11). Previously, we demonstrated that SOFAT is an important elicitor of bone breakdown when directly injected in mice, and elevated levels of SOFAT were found in inflamed gingival tissue from chronic periodontitis patients (19). Considering that periodontal disease has an important immunological feature in disease progression such as arthritis, we speculate that SOFAT could be involved in arthritic conditions.

First, we investigate the noxious effect of SOFAT on mechanical hyperalgesia response in the knee joints of mice. We demonstrated that intra-articular injection of SOFAT in the knee joint induces mechanical hyperalgesia. Specifically, this nociceptive response initiates after 3 h of injection, and the two injections assay sustained this nociceptive stage for up to 8 days. The delay to initiate the mechanical hyperalgesia response could be associated with the fact that SOFAT does not stimulate neuronal fibers directly. SOFAT induces IL-6 synthesis by synovium osteoblast, promotes osteoclast formation, and triggers the activation of the nuclear factor of activated T cells (NFAT) signal transduction pathway (11, 19). Depending on the inflammatory molecule, it can activate the nociceptor sensory neurons generating pain behavior and/or sensitize the nociceptor sensory neurons facilitating neuronal depolarization upon mechanical or thermal stimulation. Nociceptor sensory neurons sensitization accounts for chronic pain (26).

Inflammatory bone disease, such as arthritis, is characterized by massive lymphocyte infiltrate as a consequence of the initial and uncontrolled inflammatory environment (3). Subsequently, a variety of factors exacerbate inflammation, which, in turn, is responsible for an irrecoverable bone loss and pain. Persistent pain in arthritis can continue without signs of peripheral inflammation due to altered expression of TNF-α in the dorsal root ganglia with nociceptor sensory neurons activation (27). In this sense, the CIA model was conducted to investigate the expression of SOFAT in a specific model that resembles rheumatoid arthritis. SOFAT was previously described in patients with chronic periodontitis (19) and is associated with RA (11, 28) since a feedback mechanism between T cells and osteoblasts was observed.

The cellular composition of synovitis in rheumatoid arthritis includes innate immune cells and adaptive immune cells, including T and B cells. It is known that both cells are important sources of pro-inflammatory cytokines (8) as well as major RANKL producer (29). Previously data revealed that B cells, in addition to T cells, are also important cellular sources of bone destructive factors SOFAT besides RANKL (18). Thus, it is possible to suggest that SOFAT may have a role, not yet fully understood, in the inflammatory loop of RA.

Our data showed positive stain and high expression of the mRNA and protein of SOFAT in the CIA group associated with bone pits in the knee joint and aberrant leukocyte infiltrate. In agreement with this, mRNA expression of RANKL, OPG, IL-6, IL-15, and IL-1β was also found to be high in CIA. RA is a chronic autoimmune disease that attacks multiple joints (8). The RANK/RANKL/OPG is a well-known signaling pathway for osteoclast activation and the ratio between RANKL and OPG is pivotal to drive osteoclastic activation (14) as well as IL-1β and IL-6 (30). IL-6 and IL-β are innate immunity cytokines which have an important role in the pathogenesis of arthritis, by their chemotaxis and osteolytic capacities (31). For this reason, inhibitors of IL-6 and TNF-α were proposed as therapeutic strategies. However, around 50% are unresponsive to treatment as well as biologics targeting these cytokines increase the incidence of infection (8, 32). IL-15 contributes to RA physiopathology by stimulating T-cell development and survival, delaying the apoptosis of fibroblast-like synoviocytes as well as by recruiting neutrophils and lymphocytes to the inflammatory foci (17, 33, 34). A phase I-II clinical trial using a human IgG1 monoclonal antibody to target IL-15 reduced the disease activity according to the American College of Rheumatology criteria by 20% (ACR20) in 63% of patients, ACR50 in 38% of patients, and ACR70 in 25% of patients (35). This proof-of-concept study confirms experimentally in a clinical setting the relevance of IL-15 to RA disease. Considering that TNF-α, IL-1β, IL-6, and IL-15 induce pain in RA (16, 17), it is conceivable that in the CIA inflammatory context, SOFAT might also be an important hyperalgesic molecule. Of note, the hyperalgesia caused by TNF-α, IL-1β, IL-6, or IL-15 at 10 ng or a similar dose does not last in the same way as that induced by SOFAT. Thus, although these are proven hyperalgesic cytokines (16, 17), the 4 days of hyperalgesia upon a single injection of 10 ng of SOFAT is unforeseen.

Finally, we addressed the presence of SOFAT in patients previously diagnosed with RA and OA. This study presents some limitations due to the small number of subjects and the necessity for deeper understanding of the role of SOFAT in RA disease. However, this pilot study showed newsworthy findings and support the previous animal data. SOFAT protein was detected in the synovial liquid of patients diagnosed with RA for the first time. Our results showed a higher intensity of bands in immunoblotting for SOFAT in patients with RA compared to OA. ELISA assay also showed higher protein levels in RA samples than OA samples in which SOFAT was undetectable. Interestingly, patients with RA have OPG in increased levels, leading to augment in the OPG/RANKL ratio. Thus, these results reinforced the concept that SOFAT could be not only a possible therapeutic target but also a biological marker for RA and disease progression. Several new therapeutics are currently being developed on the basis of immunopathogenic insights and are being tested in trials. However, the main goal is to develop cause-directed, curative therapies, but this will not be possible without better understanding of the physiopathological mechanisms of rheumatoid arthritis.

In conclusion, this study adds a novel molecule in the complex inflammatory phenotype of RA. SOFAT induces pain *per se* and is expressed in the rheumatoid arthritis condition in an experimental mouse model and human samples.

## Data Availability Statement

The datasets generated for this study are available on request to the corresponding author.

## Ethics Statement

The studies involving human participants were reviewed and approved by the Ethics Committee in Human Research at Ribeirão Preto General Hospital and Ribeirão Preto Medical School (protocol 38156414.4.0000.5374). The patients/participants provided their written informed consent to participate in this study. This animal study was reviewed and approved by Committee on Animal Research of Faculdade São Leopoldo Mandic, Brazil (No. 2019/019) or Committee on Animal Research of Universidade Estadual de Londrina (approval under the number CEUA-UEL 28016.2014.91).

## Author Contributions

MN, JC-N, EM, and WV contributed to the study concept and design. CT-S, WD, HA, CV, MN, AR, and WV contributed to data acquisition and interpretation. CT-S, HA, JC-N, MN, and WV wrote de manuscript. All authors critically revised the manuscript and approved the submitted version.

## Conflict of Interest

The authors declare that the research was conducted in the absence of any commercial or financial relationships that could be construed as a potential conflict of interest.
